# Pannexin1 channels—a potential therapeutic target in inflammation

**DOI:** 10.3389/fcell.2022.1020826

**Published:** 2022-11-09

**Authors:** Olga M. Rusiecka, Malaury Tournier, Filippo Molica, Brenda R. Kwak

**Affiliations:** ^1^ Department of Pathology and Immunology, Faculty of Medicine, University of Geneva, Geneva, Switzerland; ^2^ Geneva Centre for Inflammation Research, Faculty of Medicine, University of Geneva, Geneva, Switzerland

**Keywords:** Panx1, inflammatory response, cell death, signaling, channels, therapeutics

## Abstract

An exaggerated inflammatory response is the hallmark of a plethora of disorders. ATP is a central signaling molecule that orchestrates the initiation and resolution of the inflammatory response by enhancing activation of the inflammasome, leukocyte recruitment and activation of T cells. ATP can be released from cells through pannexin (Panx) channels, a family of glycoproteins consisting of three members, Panx1, Panx2, and Panx3. Panx1 is ubiquitously expressed and forms heptameric channels in the plasma membrane mediating paracrine and autocrine signaling. Besides their involvement in the inflammatory response, Panx1 channels have been shown to contribute to different modes of cell death (i.e., pyroptosis, necrosis and apoptosis). Both genetic ablation and pharmacological inhibition of Panx1 channels decrease inflammation *in vivo* and contribute to a better outcome in several animal models of inflammatory disease involving various organs, including the brain, lung, kidney and heart. Up to date, several molecules have been identified to inhibit Panx1 channels, for instance probenecid (Pbn), mefloquine (Mfq), flufenamic acid (FFA), carbenoxolone (Cbx) or mimetic peptides like ^10^Panx1. Unfortunately, the vast majority of these compounds lack specificity and/or serum stability, which limits their application. The recent availability of detailed structural information on the Panx1 channel from cryo-electron microscopy studies may open up innovative approaches to acquire new classes of synthetic Panx1 channel blockers with high target specificity. Selective inhibition of Panx1 channels may not only limit acute inflammatory responses but may also prove useful in chronic inflammatory diseases, thereby improving human health. Here, we reviewed the current knowledge on the role of Panx1 in the initiation and resolution of the inflammatory response, we summarized the effects of Panx1 inhibition in inflammatory pathologies and recapitulate current Panx1 channel pharmacology with an outlook towards future approaches.

## Introduction

Intercellular signaling is a crucial biological process in all organisms. This ability to receive, process and send signaling stimuli allows cells to communicate with their environment and with each other. The exchange of molecules between the extra-, intra- and intercellular compartments is crucial to maintain tissue homeostasis but also leads to the propagation of pathological stimuli (Herve and Derangeon, 2013). One way to transmit signals between two neighboring cells is through specialized membrane contacts–gap junctions (GJs)—built of connexins (Cxs) (Herve and Derangeon, 2013; Delmar et al., 2018). In the last decade, a new family of channel-forming proteins, pannexins (Panxs), has emerged (Panchin et al., 2000). Despite a similar topology, Panxs and Cxs differ from each other in terms of sequence, channel assembly and their ability to form intercellular channels (Penuela et al., 2013).

## The pannexin protein family

Panxs form a family of channel-forming proteins described in 2000 (Panchin et al., 2000). They share topological similarities with Cxs and innexins (Inxs). The selective expression pattern of these channels as well as their different gating properties along with different molecular permeability create a diverse metabolically coupled network in multicellular organisms (Shestopalov and Panchin, 2008).

The Panx family comprises three proteins: Panx1 (with a predicted molecular weight of 47,6 kDa), Panx2 (74,4 kDa) and Panx3 (44,7 kDa). All three proteins display around 50%–60% of sequence similarity in humans (Panchin et al., 2000; Sosinsky et al., 2011). They are composed of four transmembrane domains (TM), one intracellular loop (IL) and two extracellular loops (EL) with a C- and an N-terminus (CT and NT) directed to the cytosol. Panxs are characterized by their ability to oligomerize into single-membrane channels called pannexons. The glycosylation site located on the first (Panx2, Panx3) and second (Panx1) EL was long thought to prevent the docking of two adjacent channels and the formation of a channel that spans two plasma membranes, similar to the ones found in GJs (Dahl and Keane, 2012). As such, the main role of Panx channels is to mediate intercellular signaling allowing for the exchange of ions and small metabolites (up to 1.5 kDa in size) between the intra- and extra-cellular compartments (Shestopalov and Panchin, 2008; D'Hondt et al., 2011; Molica et al., 2018). They have also been reported to contribute to the intracellular Ca^2+^ homeostasis (Shestopalov and Panchin, 2008). This single-membrane channel dogma was recently challenged by Palacios-Prado et al. (2022) showing electrophysiological recordings of Panx1-mediated GJ-like direct cell-cell communication in transfected HeLa cells and in a human oligodendroglioma cell line naturally expressing Panx1. Whether primary cells and other cell lines show similar properties remains to be proven in the coming years.

The glycosylation of Panx1 at the position N254 is known to regulate trafficking and cellular location of pannexons (Boassa et al., 2007; Boassa et al., 2008; Penuela et al., 2009; Sandilos and Bayliss, 2012). The non-glycosylated and high-mannose glycosylated forms (Gly0 and Gly1, respectively) are mostly found in intracellular compartments, while the fully processed form (Gly2) is inserted into the plasma membrane. The half-life of Panx1 has been reported to be comparable to the one of the P2X7 purinergic receptor (P2X7R) and estimated around 54 h (Boassa et al., 2007; Boassa et al., 2008; Gehi et al., 2011; Sandilos and Bayliss, 2012).

Panxs exhibit different expression patterns. While Panx1 can be found in the vast majority of mammalian tissues, the two other Panx appear to have a more restricted expression pattern (Jiang and Penuela, 2016). Panx2 is mostly found in the central nervous system (CNS) in humans and rodents (Le Vasseur et al., 2014). Some studies have shown very low Panx2 mRNA levels in the eye, thyroid, kidney, liver or intestine (Penuela et al., 2013; Le Vasseur et al., 2014). Panx3 was found in adult bones, cartilage and skin (Penuela et al., 2008; Sosinsky et al., 2011). It was also detected in murine joints in the paws and the inner ear cartilage as well as in osteoblasts and synovial fibroblasts (Penuela et al., 2013). It is likely that the number of tissues in which Panx2 and Panx3 expression is reported will grow in the coming years as better antibodies become generally available and proteomic techniques are increasingly used.

## Panx1 channel properties and physiological function

Although it has been initially reported that functional Panx1 channels consist of six monomeric units (Shestopalov and Panchin, 2008), recent structure analyses by cryo-electron microscopy (cryo-EM) have undoubtedly revealed that Panx1 channels are heptameric (Deng et al., 2020; Jin et al., 2020; Michalski et al., 2020; Qu et al., 2020). Moreover, these studies revealed the size of the pore over 15 Å within the TM and NT funnel formation, and the secondary structure of Panx1 monomers to consist of a helical NT, 4 TM helices, three helical segments and a β strand in the EL1 and five helices in the CT (Kuzuya et al., 2022).

Panx1 channels allow for the flux of small molecules and metabolites up to 1.5 kDa (e.g., glutamate, arachidonic acid, nucleotides including ATP and UTP) and ions between intra- and extra-cellular compartments (Shestopalov and Panchin, 2008; Sandilos and Bayliss, 2012; Sandilos et al., 2012; Molica et al., 2015). Such molecules can mediate auto-, endo- and paracrine signaling and give rise to downstream signaling and cellular responses (Crespo Yanguas et al., 2017; Lee et al., 2018). So far, Panx1 channels are mainly recognized for their regulated non-vesicular release of ATP. Inhibition of Panx1 channel activity notably affects ATP-related signaling in cells (Dahl and Keane, 2012). Panx1 channels can also pass cationic and anionic dyes (YO-PRO-1 and Lucifer Yellow, for instance), which are commonly used to study Panx1 channel activity (Shestopalov and Panchin, 2008; Penuela et al., 2013; Crespo Yanguas et al., 2017). Channel function is abolished by cytosolic acidification (Shestopalov and Panchin, 2008), the negative feedback exerted by ATP (Qiu and Dahl, 2009) or by pharmacological blocking (Penuela et al., 2013).

A crucial role for Panx1 channels in signal transduction is further underlined by their role in Ca^2+^ fluxes (Shestopalov and Panchin, 2008). In fact, the open Panx1 channel contributes to an influx of Ca^2+^. Calcium wave propagation is triggered in response to ATP-mediated purinergic receptor stimulation (P2XR, P2YR), which further increases inositol 1,4,5- triphosphate (IP_3_) concentration, thereby triggering the release of Ca^2+^ from the ER (Penuela et al., 2013). High intracellular Ca^2+^ leads, in turn, to Panx1-mediated ATP release which further propagates those signals. Calcium waves can be also transferred between cells *via* the classical GJ-mediated route (Shestopalov and Panchin, 2008). Furthermore, Panx1-overexpressing cells exhibit higher endoplasmic reticulum (ER) calcium permeability with increased calcium storage in these organelles upon Panx1 deletion (Shestopalov and Panchin, 2008; Lee et al., 2020).

Panx1 channels open in response to a variety of physiological and pathological stimuli originating from the inside or the outside of the cell (Shestopalov and Panchin, 2008). In most mammalian cell types, Panx1 channels can be activated upon mechanical stress (Bao et al., 2004; Lopez et al., 2021), membrane depolarization (D'Hondt et al., 2011), high extracellular K^+^ (Molica et al., 2015) and intracellular Ca^2+^ concentrations (D'Hondt et al., 2011), oxygen/glucose deprivation as occurs during ischemia (Thompson et al., 2006; Kawamura et al., 2010; Kirby et al., 2021; Seo et al., 2021), Src-mediated phosphorylation (following ionotropic-, chemokine- and glycoprotein receptor stimulation) (Weilinger et al., 2012; Molica et al., 2019) and in response to stimulation of purinergic receptors with extracellular ATP, which leads to “ATP-induced ATP release” (Shestopalov and Panchin, 2008). Finally, Panx1 channels are activated in an irreversible manner by caspase-dependent cleavage of the CT in cells undergoing apoptosis (Chekeni et al., 2010; Sandilos et al., 2012). Studies on human Panx1 showed that in the native conformation, the channel is inhibited by the interaction with its own CT domain *via* a “ball-and-chain” mechanism (Sandilos et al., 2012; Chiu et al., 2014). A specific cleavage at the amino acids 376–379 within the CT region disrupts this interaction leaving the channel in a permanently open state (Sandilos et al., 2012).

Panx1 has been recognized as one of the largest-pore forming channels in the human body (Dahl and Keane, 2012). Electrophysiological properties of Panx1 channels were initially studied in *Xenopus* oocytes and later detailed in human and rodent cell lines (Shestopalov and Panchin, 2008; Chiu et al., 2014). Although Panx1 channels are generally considered as non-selective, permeating negatively and positively charged molecules, an anion selectivity has been reported in several independent studies (Ma et al., 2012; Romanov et al., 2012; Chiu et al., 2014; Michalski et al., 2020). Mutating Trp74 or Arg75 of the frog Panx1 has been shown to disrupt anion selectivity (Michalski et al., 2020). It should be kept in mind however that Panx1 channel gating is insensitive to the removal of extracellular Ca^2+^, which distinguishes it from Cx hemichannels (Shestopalov and Panchin, 2008; Sandilos et al., 2012). The channel can open and close at a broad range of voltages (−100 to +100 mV), however, at the resting state, its permeability to ATP remains very low (Lopez et al., 2021). Panx1 channels have multiple conductance states with the maximal reaching 500 pS (Sandilos and Bayliss, 2012; Kuzuya et al., 2022). The physiological stimuli mentioned above trigger the large conductance configuration of the Panx1 channel (Wang et al., 2014), while the voltage-dependent activation in the absence of extracellular K^+^ induces a low conductance state (50 pS) with no permeability to ATP (Wang et al., 2014).

The ubiquitous expression pattern of Panx1 suggests that this protein might be involved in a variety of physiological cellular processes. During the early development, Panx1 regulates the proliferation and differentiation of dermal fibroblasts, keratinocytes, and myoblasts in skeletal muscles as well as osteoblasts and neuronal progenitors (Celetti et al., 2010; Penuela et al., 2013; Lee et al., 2018). For instance, Panx1 channels were found at the surface of skeletal muscle myoblasts and satellite cells. Surprisingly, in differentiating muscle cells, Panx1 was highly upregulated (Suarez-Berumen et al., 2021). Although muscle regeneration does occur in Panx1-deficient mice, the newly formed myofibers are significantly smaller and their amount is reduced. It appeared that the ATP released through Panx1 channels is essential for myoblast migration and fusion. Hence, the role of Panx1 in skeletal muscle differentiation relates to its downstream signaling and crosstalk with the P2R pathway that supports myocyte fusion (Riquelme et al., 2013). The ATP released by Panx1 channels in skeletal muscle cells is necessary for the potentiation of contraction (Riquelme et al., 2013). Panx1 is also a crucial regulator in cardiovascular physiology. Indeed, Panx1 channels in vascular smooth muscle cells (SMCs) are key players in α-adrenergic receptor-stimulated vasoconstriction of resistance arteries, which controls blood pressure (DeLalio et al., 2018). In addition, Panx1 channels appeared to be involved in endothelial cell (EC)-dependent regulation of arterial vasodilation (Gaynullina et al., 2015; Lillo et al., 2021). Finally, a role for Panx1 in platelet activation regulating thrombosis and hemostasis has been described (Taylor et al., 2014; Molica et al., 2015; Molica et al., 2019). Thus, Panx1 participates in a plethora of physiological processes. In addition, these channels regulate the course of pathophysiological processes including inflammation and cell death (Dahl and Keane, 2012; Koval et al., 2021).

## Panx1 channels in the inflammatory response

**FIGURE 1:**
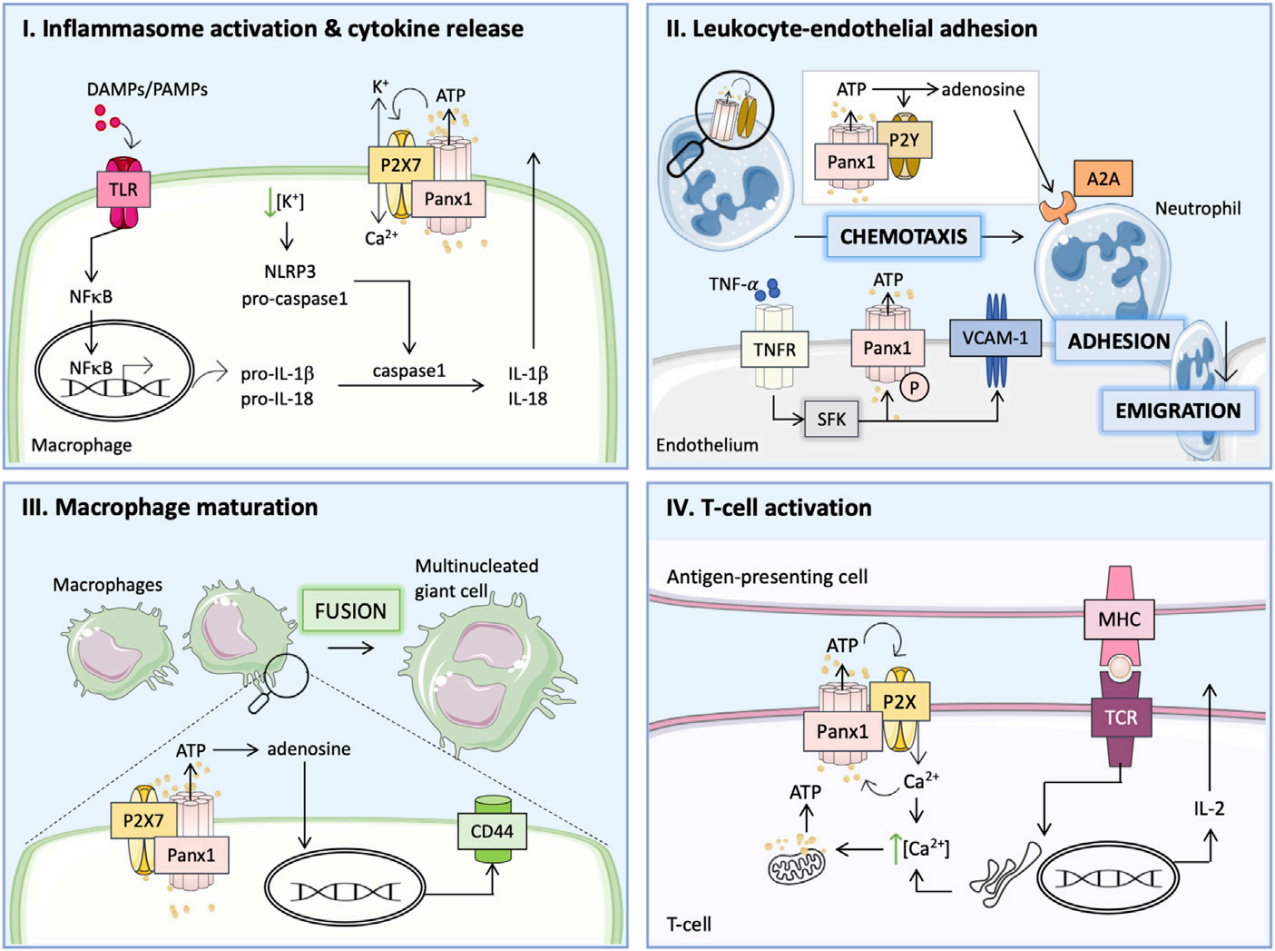
Panx1 participates in the inflammatory response *via* distinct pathways. (I) Damage-associated molecular patterns (DAMPs) or pathogen-associated molecular patterns (PAMPs) act *via* Toll-like receptors (TLRs) to activate nuclear factor κB (NF-κB), which controls the transcription of pro-inflammatory cytokines and the immature forms of interleukin-1β (pro-IL-1β) and pro-IL-18. Together with Panx1, P2X7 receptors mediate ATP release and affect ion flux, which enhances activation of the NLRP3 inflammasome and the formation of mature caspase-1 leading to the cleavage of pro-ILs. (II) TNFα binds to its receptor (TNFR) and activates the Panx1 channel by SFK-mediated phosphorylation (P) and increases the expression of vascular cell adhesion molecule-1 (VCAM-1). ATP released *via* Panx1 channels attracts neutrophils to adhere and emigrate through the endothelial layer into the injured tissue. The interaction between Panx1 and P2Y receptors on neutrophils enhances ATP release, which subsequently acts as a chemotactic stimulus. ATP conversion to adenosine provides necessary inhibitory signals *via* A2A receptor stimulation leading to the resolution of inflammation. (III) During chronic inflammation, ATP released by Panx1 channels is further processed into adenosine and induces the expression of surface molecules (e.g., CD44) essential for macrophage fusion to multinucleated giant cells. (IV) T-cell activation requires the formation of immune synapses, which allows for interaction between T-cell receptor (TCR) and major histocompatibility complex (MHC) on the antigen-presenting cells. Ca^2+^ influx upon TCR stimulation triggers mitochondrial-dependent ATP production. Ca^2+^ release from the ER further increases the intracellular Ca^2+^ concentration inducing Panx1 channel opening. The ATP released through Panx1 channels stimulates P2XR in an autocrine manner and generates positive feedback on TCR stimulation, thus enhancing IL-2 production and T-cell proliferation.

Inflammation is an essential process providing a physiological defense and repair mechanism in case of injury or infection. Thanks to the proper clearance of dying cells, their debris and potential pathogens, inflammation helps to maintain the homeostatic balance. Short-term (acute) inflammation promotes tissue regeneration in most organs. However, a chronic inflammatory response is a malfunction associated with several pathologies. The different stages of the inflammatory process (acute vs. chronic and innate vs. adaptive) as well as their time course have been described in detail elsewhere (Ley, 2003; Ley et al., 2007; Geissmann et al., 2010; Kumar et al., 2017; Soehnlein et al., 2017; Adrover et al., 2019; Margraf et al., 2019). In this review, we will focus on the contribution of Panx1 channels in key stages of the inflammatory response, which is also summarized in Figure 1 (Woehrle et al., 2010; Makarenkova and Shestopalov, 2014; Lohman et al., 2015; Crespo Yanguas et al., 2017; Makarenkova et al., 2018).

During the initial phase of inflammation, the binding of damage-associated molecular patterns (DAMPs) or pathogen-associated molecular patterns (PAMPs) to Toll-like receptors (TLRs) induces NFκB-dependent expression of immature cytokines (pro-IL-1β and pro-IL-18) (Crespo Yanguas et al., 2017). Next, the activation of the NLRP3 inflammasome induces caspase-1, which cleaves the immature ILs leading to the release of active IL-1β and IL-18, thereby augmenting the inflammatory response (Crespo Yanguas et al., 2017). Panx1 was found to participate in the second step of this mechanism by stimulating P2X7R *via* ATP release, which induces Ca^2+^ influx with simultaneous K^+^ efflux and promotes “ATP-induced ATP release” and NLRP3 inflammasome activation as shown in Figure 1, panel I (Pelegrin and Surprenant, 2006; Silverman et al., 2009; Crespo Yanguas et al., 2017). As such, Panx1 channels were shown to be essential for the release of mature IL-1β following lipopolysaccharide (LPS)-induced stimulation of P2X7R in murine macrophages, human THP-1 monocytes and human alveolar macrophages (Pelegrin and Surprenant, 2006). The involvement of Panx1 in inflammasome activation was further demonstrated in macrophages, microglia, neurons and astrocytes (Silverman et al., 2009; Makarenkova et al., 2018). Indirectly, the Panx1 channel is thus thought to mediate macrophage maturation to the M1 phenotype responsible for boosting the inflammatory response induced by this cytokine release (Makarenkova and Shestopalov, 2014). While in monocytes/macrophages low intracellular K^+^ promotes inflammasome assembly, in neuronal cells high intracellular K^+^ was associated with inflammasome activation *via* Panx1 (Silverman et al., 2009). Regarding the latter, Panx1 was also shown to interact with inflammasome components such as P2X7R and caspase-1 (Silverman et al., 2009).

Panx1 is also a major contributor to leukocyte activation and chemoattraction to injured areas (Bao et al., 2013). During the acute inflammatory response, tumor necrosis factor-α (TNFα)-activated endothelium releases signaling molecules that attract leukocytes (primarily neutrophils) and boost the inflammatory response (Dubyak, 2002). Cytokine-induced upregulation of adhesion molecules on the surface of ECs mediates the adhesion of leukocytes (Zerr et al., 2011). Selectins (P-selectin and E-selectin) are required for the initial attachment of inflammatory cells and their rolling over the endothelium. Firm adhesion is mediated by members of the immunoglobulin family: vascular cell adhesion molecule-1 (VCAM-1) and intercellular adhesion molecule-1 (ICAM-1) (Zerr et al., 2011). Activated leukocytes express α4 and β2 integrins that bind to VCAM-1 and ICAM-1, respectively (Zerr et al., 2011; Fotis et al., 2012). This interaction allows for firm adhesion of inflammatory cells to ECs, a step that is necessary for their extravasation (Zerr et al., 2011; Fotis et al., 2012; Bao et al., 2013). Endothelial Panx1 mediates leukocyte-EC adhesion by releasing ATP in response to TNFα. This cytokine binds to its receptor (TNFR) triggering a downstream signaling cascade through SFK that phosphorylates Panx1 at Y198 and opens the channel (Bao et al., 2013; Lohman et al., 2015). Open Panx1 channels release ATP, which acts in an autocrine manner to stimulate P2Y6R. Purinergic signals are known to contribute to NFκB-mediated upregulation of endothelial P-selectin, VCAM-1 and ICAM-1 expression. Importantly, the induction of VCAM-1 in response to cytokine treatment is abolished in Panx1-deficient mice, an effect that can be reversed by ATP (Lohman et al., 2015). Furthermore, genetic ablation of Panx1 and P2Y1R or Panx1 and P2Y6R abolished inflammatory cell recruitment as well as expression of endothelial adhesion molecules, further underlining the importance of this crosstalk in the process of leukocyte adhesion (Bao et al., 2013). Of note, TNFα-dependent activation of NFκB also enhances Panx1 expression in ECs (Yang et al., 2020). Panx1 channels were demonstrated to facilitate Ca^2+^ influx in activated ECs, thereby further contributing to IL-1β production and release (Yang et al., 2020). A recent study also demonstrated a potential role for Panx1 in the increase of endothelial permeability in the TNFα-induced inflammatory response (Maier-Begandt et al., 2021). As described above, TNFR stimulation results in SFK-dependent Panx1 phosphorylation, and to the liberation of ATP molecules that are further converted to adenosine by the combined action of ectonucleoside triphosphate di-phosphohydrolase (CD39) (ecto-apyrase) and 5′-nucleotidase (CD73). Subsequent induction of A2A/A2B adenosine receptors evokes positive effects on transient receptor potential vanilloid 4 (TRPV4). This channel mediates Ca^2+^ influx and increases endothelial permeability partially by downregulation of tight junction proteins (Maier-Begandt et al., 2021). Interestingly, these effects were much stronger in the venous than in the arterial endothelium, which correlates with an increased level of CD39 in the venous endothelium (Maier-Begandt et al., 2021).

Neutrophil chemotaxis depends on positive stimulation at the cell’s front and a negative (inhibitory) one at the back (Bao et al., 2013). ATP release through Panx1 was shown to act as a driving signal at the front of the cell by autocrine stimulation of P2YRs on the surface of the neutrophils (Dubyak, 2002; Bao et al., 2013; Crespo Yanguas et al., 2017). Purinergic receptor stimulation amplifies the signals and induces a cell polarization (Bao et al., 2013). At the same time, ATP is further converted by CD39 and CD73 to adenosine, which provides the inhibitory signal at the back of the cell *via* A2A adenosine receptors and the PKA/cAMP downstream signaling (Dubyak, 2002; Bao et al., 2013; Crespo Yanguas et al., 2017). Adenosine was also shown to act *via* A3-type receptors, favoring the excitatory signals at the front of polarized neutrophils (Chen et al., 2006; Bao et al., 2013). The proposed mechanisms explaining the role of Panx1 in neutrophil chemoattraction are presented in Figure 1, panel II (Bao et al., 2013). Hence, Panx1 constitutes a key player in neutrophil migration contributing to the inflammatory response (Bao et al., 2013; Makarenkova and Shestopalov, 2014).

One of the hallmarks of chronic inflammation is the formation of giant multinucleated cells by macrophage fusion (McNally and Anderson, 2005, 2011). Multinucleation is a key step in osteoclast differentiation, as mononucleated macrophages cannot resorb bone efficiently. Multinucleation may also be essential in the differentiation of giant cells, which form in tissues in response to foreign particles (Vignery, 2005). This process can be triggered in response to numerous cytokines released by inflammatory cells (e.g., GM-CSF, IL-4, IL-13, IL-6) (Lemaire et al., 2011). Although the exact mechanism remains elusive, it is recognized that macrophage fusion is mediated by several membrane proteins like CD44 or β-integrin receptors (Lemaire et al., 2011). Panx1 has been proposed to participate in macrophage fusion together with P2X7R by releasing ATP that is rapidly converted to adenosine, which promotes macrophage fusion by upregulating CD44 expression at the cell membrane as illustrated in Figure 1, panel III (Lemaire et al., 2011). A study performed with pharmacological inhibitors as well as genetic knock-out animals evidenced that while both the Panx1 channel and the P2X7R appeared to be essential for macrophage fusion they likely act independently *via* distinct pathways (Lemaire et al., 2011).

Interaction between T-cells and antigen-presenting cells (APCs) promotes the adaptive immune response (Tai et al., 2018). This process takes place within the immune synapses (Tai et al., 2018). T-cell receptor (TCR) interaction with the major histocompatibility complex (MHC) on the APC causes a Ca^2+^ influx that triggers mitochondrial-dependent ATP production within the T-cell (Schenk et al., 2008). Elevated intracellular Ca^2+^ activates Panx1 channels which, in conjunction with P2XRs, mediate ATP release and thereby the autocrine stimulation of the TCR as shown in Figure 1, panel IV (Schenk et al., 2008; Woehrle et al., 2010). This positive feedback loop involving Panx1 channels was first observed in stimulated mouse CD4^+^ and Jurkat cells and was then confirmed in the human primary T-cells (Woehrle et al., 2010). Thus, ATP provides an amplifying signal for the T-cell activation (Woehrle et al., 2010). Activation of the TCR results in translocation of Panx1 with P2X1R and P2X4R to the immune synapse, where they contribute to this Ca^2+^-dependent ATP signaling (Woehrle et al., 2010). Similar interactions were observed between Panx1 and P2X7R (Woehrle et al., 2010). P2XRs contribute to nuclear factors of activated T-cells (NFAT) activation and subsequent IL-2 release, thereby promoting the T-cell proliferation (Woehrle et al., 2010; Crespo Yanguas et al., 2017). Blocking Panx1 channels reduced Ca^2+^ influx and IL-2 expression in human and mouse primary T-cells demonstrating its contribution to the immune response (Woehrle et al., 2010). Finally, the ATP released from dying cells through Panx1 channels acts as a “find me” signal for phagocytic macrophages mediating the cell clearance and is an integral process of the resolution of the inflammatory response (Chekeni et al., 2010; Medina and Ravichandran, 2016; Narahari et al., 2021).

In conclusion, ATP release through Panx1 channels appeared to enhance or promote inflammation at multiple stages of the process. Thus, selective inhibition of Panx1 channels may not only limit acute inflammatory responses but may also prove useful in chronic inflammatory diseases, thereby improving human health.

## Panx1 channels in cell death

Activation of Panx1 channels has been associated with the onset and propagation of cell death (Penuela et al., 2013). As described above, Panx1 forms large and non-selective channels, and their irreversible opening is detrimental (D’Hondt et al., 2011). Prolonged opening of Panx1 channels leads to the liberation of signaling molecules that are recognized as ‘danger signals’ and trigger inflammatory responses (D’Hondt et al., 2011). ATP can also be released from the injured cells undergoing necrosis as well as from infiltrating leukocytes through Panx1 channels. ATP release leads to an important loss of energy and stimulates necrotic cell death (Makarenkova and Shestopalov, 2014). Already in 2006, ischemia-like conditions such as oxygen/glucose deprivation have been shown to open a large conductance (530 pS) channel in neurons (later identified as Panx1), which is involved in excitotoxicity and neuronal death during stroke (Thompson et al., 2006; Thompson and Macvicar, 2008; Weilinger et al., 2012; Weilinger et al., 2016). Panx1 was also proposed to participate in the crosstalk between mixed lineage kinase-like (MLKL) and Rab27 protein (Rab GTPase) leading to the generation and shedding of small extracellular vesicles (EVs) during early stages of necroptosis–a programmed form of necrotic cell death that plays an important role in several pathologies (Douanne et al., 2019).

In addition to the contribution of Panx1 to the induction of necrotic signals, many studies have shown an involvement of Panx1 in apoptosis, a programmed, energy-dependent form of cell death, which represents a vital component of homeostasis in maintaining the cellular turnover (Elmore, 2007; Qu et al., 2011; Crespo Yanguas et al., 2017). Cells that undergo apoptosis display characteristic shrinkage, chromatin condensation and karyorrhexis. The blebbing of plasma membranes and fragmentation of cell content into apoptotic bodies (budding) are also apoptosis-related features. Of note, the cellular integrity is maintained during this process, and thus apoptosis is considered as an immunologically silent form of cell death (Elmore, 2007). The morphological changes within cells are mediated by proteolytic caspase cleavage. Caspases can be activated *via* two distinct pathways. Intrinsically, caspase activation can be mediated by mitochondria and families of pro- and anti-apoptotic proteins (e.g., Bax and Bcl-2, respectively) (Crespo Yanguas et al., 2017; Huang et al., 2021). The extrinsic pathway is related to the plasma membrane receptor stimulation by extracellular ligands (e.g., Fas ligand or TNFα). Panx1 has been shown to be involved in different stages of the apoptotic cell death (Qu et al., 2011; Crespo Yanguas et al., 2017). First, Chekeni and colleagues reported that Panx1 channel activation during early apoptotic stages mediates the ATP/UTP “find me” signal release that attracts phagocytic cells to the injured area and mediates clearance of the cellular debris (Chekeni et al., 2010). Both pharmacological blocking as well as genetic knockdown of Panx1 reduced ATP release and subsequent monocyte recruitment (Chekeni et al., 2010; Sandilos et al., 2012). The progression of apoptosis was however not impaired in Panx1-deficient mice. As such, the ATP/UTP signaling is mostly crucial during the early stages of the apoptotic cell death (Chekeni et al., 2010). Further studies revealed that Panx1 is a target for both caspase 3 and caspase 7 and that it possesses a specific cleavage site located within its C-terminus, i.e., D378 and D379 for the mouse and human sequence, respectively (Dahl and Keane, 2012; Sandilos et al., 2012; Yang et al., 2015; Boyce et al., 2018). Truncation of the C-terminal tail leaves the Panx1 channel permanently open this way facilitating cell death (Qu et al., 2011; Dahl and Keane, 2012). Interestingly, a recent study on a rat model of spinal cord injury showed an upregulation of Bax and caspase-3 with a simultaneous reduction in Bcl-2 level in neurocytes overexpressing Panx1, which resulted in increased apoptosis (Huang et al., 2021). Consequently, Panx1-deficient cells exhibited the opposite response. The same authors showed the contribution of Panx1 to Ca^2+^ overload-induced ER stress and apoptosis of spinal neurocytes *via* an ER-related pathway (Huang et al., 2021).

Independent of the molecular mechanism involved, the above paragraphs underline the crucial role of Panx1 channels in the regulation of inflammation and cell death. Altogether, this suggests that blocking Panx1 channels may alter the outcome of diseases that critically depend on these processes.

## The challenge of specific channel inhibition: Panx1 pharmacology

Over the years, Panx1 channels were shown to be inhibited by malaria drugs, chloride channel blockers, ligands of purinergic receptors and inflammasome inhibitors (Dahl et al., 2013; Navis et al., 2020). This variety in drug classes may be (partially) explained by the wide range of cellular processes in which Panx1 participates in concert with other proteins, or by the potential similarity of the epitopes between Panx1 and other targets. Nevertheless, a vast majority of inhibitors that have been used to study Panx1 channels up till now exhibit a rather poor specificity (Dahl et al., 2013). The following paragraphs contain a brief description of commonly used pharmacological Panx1 channel inhibitors.

The non-steroidal anti-inflammatory drug Flufenamic acid (FFA) is a Fenamate-derivative chloride channel blocker that was first shown to decrease Cx hemichannel currents (Dahl et al., 2013). In 2005, Bruzzone and colleagues reported a modest inhibition of Panx1 channels expressed in *Xenopus* oocytes by FFA without changes in the kinetics or in the voltage gating of the channel (Bruzzone et al., 2005). FFA is however known to have gastrointestinal side effects, which together with its poor specificity for Panx1 channels, limits its potential to re-purpose this drug for Panx1-mediated disease processes (Dolmatova et al., 2012; Molica et al., 2019).

Panx1 channel inhibition by the anti-malaria drug 4-quinolinemethanol, known as Mefloquine (Mfq), was first reported in 2008 and further confirmed in follow-up studies (Iglesias et al., 2008; Iglesias et al., 2009; Dahl et al., 2013). In fact, its parental molecule quinine is also able to block Panx1 channel currents but to a lesser extent (Dahl et al., 2013). Mfq was found to inhibit Panx1 channel-mediated platelet aggregation (Molica et al., 2019), Phenylephrine-induced vasoconstriction (Billaud et al., 2011) and reduced post-ischemic middle cerebral artery occlusion (MCAO)-induced injury in mice (Cisneros-Mejorado et al., 2015). However, Mfq cannot be considered a selective Panx1 channel antagonist as it has been shown to also block GJ channels and the P2X7R-mediated dye uptake (Cruikshank et al., 2004; Suadicani et al., 2006; Iglesias et al., 2009; Dahl et al., 2013). Other serious disadvantages of this remedy against malaria are its psychiatric side effects such as anxiety, paranoia, psychosis and anterograde amnesia (Molica et al., 2019).

The Enoxolone derivative–Carbenoxolone (Cbx) is a Food and Drug Administration (FDA)-approved drug against gastric ulcers, which was already in 1986 shown to block Cx43 channels (Dahl et al., 2013). Several years later, Bruzzone and colleagues demonstrated its inhibitory effect on Panx1 channels and since then it has been commonly used in the Panx1 research field (Bruzzone et al., 2005; Dahl et al., 2013; Molica et al., 2019). The inhibition of Panx1 channels by Cbx displays a concentration-dependent response with an IC_50_ at 5 μM (Bruzzone et al., 2005; Ma et al., 2009). It has been shown to affect the voltage dependency of Panx1 channels (Dahl and Keane, 2012; Dahl et al., 2013). Recent cryo-EM analysis of Panx1 channels demonstrated that Cbx triggers allosteric inhibition by clustering in the groove between the Panx1 EL1 and EL2 domains, thereby fixing its closed conformation (Michalski and Kawate, 2016; Michalski et al., 2020). Despite this defined binding to Panx1 EL domains, Cbx also exhibits inhibitory effects on P2X7R (Ma et al., 2009) and Cx channels - although the latter requires a higher concentration than used for Panx1 channels (Chekeni et al., 2010; Dahl et al., 2013). Despite its poor selectivity, it was shown that Cbx-induced Panx1 channel inhibition mitigated cancer metastasis in mice (Freeman et al., 2019), reduced platelet aggregation (Molica et al., 2019) and suppressed the activation of the NLRP3 inflammasome (Chen et al., 2020). It also exhibited protective effects in various types of ischemic injury e.g., acute kidney ischemia/reperfusion (I/R) injury (Jankowski et al., 2018), lung I/R (Sharma et al., 2018) or stroke (Good et al., 2018b). Still, the side effects triggered by Cbx treatment, including sodium retention or hypokalemia, are noteworthy (Molica et al., 2019).

The Panx1 channel blocking capacities of 4-(dipropylsulfamoyl)benzoic acid, known as Probenecid (Pbn), outperforms the ones of Cbx and FFA (Chekeni et al., 2010; Dahl and Keane, 2012; Dolmatova et al., 2012). Pbn is a commonly used medication to prevent gout and gouty arthritis. It belongs to the uricosurics class of drugs, i.e., it increases renal uric acid secretion by inhibiting the reabsorption of uric acid through the organic anion transporter in the proximal tubules (Ma et al., 2009; Dahl and Keane, 2012). Studies on *Xenopus* oocytes revealed the Panx1 channel blocking potential of Pbn and the lack of action of this drug on Cx channels (Silverman et al., 2008; Sandilos and Bayliss, 2012). The slower kinetics of Panx1 channel blocking by Pbn, when compared to Cbx, suggest a distinct mechanism of action of this compound (Ma et al., 2009). Panx1 channel inhibition by Pbn was shown to diminish caspase-1 activation and inflammasome activation in many cell types, this way contributing to inhibition of the inflammatory response (Dahl and Keane, 2012; Xiong et al., 2014; Sharma et al., 2018; Good et al., 2021). As inflammation is the core pathology of gout, inhibition of Panx1 channels may be an additional mechanism by which Pbn exerts its beneficial therapeutic actions. In summary, Pbn is a powerful Panx1 channel inhibitor, but its selectivity is questionable due to additional effects on the anion transporter and P2X7R, and its usability thus remains under debate (Ma et al., 2009; Willebrords et al., 2017).

The ATP released through Panx1 channels stimulates P2X7Rs and triggers a positive feedback loop in cell types with expression of both molecules (Dahl and Keane, 2012; Dahl et al., 2013). The opening of the Panx1 channel within the Panx1/P2X7R complex is possibly linked to conformational changes of the purinergic partner (Ma et al., 2009). However, in cells lacking the P2X7R, the same concentration range of exogenous ATP inhibits Panx1 current reaching 90% of the Cbx-induced effects (Ma et al., 2009). The existence of two functionally different Panx1 channel populations has been postulated: the first one involved in the P2X7R complex, which would enforce a conformation of Panx1 channels not accessible for direct ATP binding (but still sensitive for Cbx and Pbn), and the second one independent from P2Rs and thus available for direct ATP inhibition (Ma et al., 2009). On the other hand, potential differences in the affinity of ATP for P2X7R and Panx1 allow for better control of Panx1 channel permeability by ATP. This could be considered as a protective mechanism avoiding complete ATP depletion and thus protecting the cell from death (Qiu and Dahl, 2009; Dahl and Keane, 2012; Dahl et al., 2013). Such a negative feedback loop was also reported by Dahl and Qiu who observed transient activation of Panx1 channels with extracellular ATP in *Xenopus* oocytes followed by the inhibitory response (Qiu and Dahl, 2009; Dahl et al., 2013). The Panx1-ATP relation was shown as strictly concentration-dependent where lower ATP concentrations activate the Panx1 channel and higher concentrations suppress its activity. The exact mechanism of the ATP binding and activation/inhibition has not yet been revealed, but it likely involves steric interactions with the channel (Qiu and Dahl, 2009). Although Panx1 does not possess any of the canonical ATP binding sites, mutagenesis studies indicate the involvement of positively charged amino acids within the Panx1 ELs (Qiu and Dahl, 2009). Of further note, Panx1 channels are insensitive to intracellular ATP concentrations (Ma et al., 2009).

ATP analogs also possess Panx1 channel blocking capacities. Indeed, Brilliant Blue G (BBG) or benzoyl-benzoyl-ATP (BzATP) abolished ATP release from *Xenopus* oocytes and Panx1-mediated dye uptake in erythrocytes (Qiu and Dahl, 2009; Dahl et al., 2013). BBG was further shown to prevent inflammasome activation in neurons *via* blocking of P2X7R (Dahl and Keane, 2012) and it protected against cerebral ischemia-reperfusion injury in rats (Chu et al., 2012). BBG belongs to a family of dyes that have wide applications in the food industrial field (Ferreira et al., 2016). Its derivative BB-FCF (also named FD&C Blue No.1 or E133) is one of the most applied food dyes. BB-FCF was also shown to modulate the activity of Panx1 channels; it inhibited Panx1 currents in *Xenopus* oocytes, ATP release from platelets and it decreased collagen-induced aggregation of human platelets (Molica et al., 2015; Ferreira et al., 2016). *In vivo* (oral) administration of BBG is considered safe, including for humans (Ferreira et al., 2016). Still, their application to study Panx1 function has been very limited so far and not much is known about the pharmacological selectivity of BB-based dyes for Panx1 channels (Qiu and Dahl, 2009; Dahl and Keane, 2012).

Spironolactone is an essential drug to treat resistance hypertension by acting on mineralocorticoid receptor (MR) NR3C2 (Sica, 2015). This drug has been shown to possess a Panx1 channel inhibitory capacity in an unbiased small molecule screening study (Good et al., 2018a). Spironolactone was shown to decrease Panx1-mediated α-adrenergic vasoconstriction in human arterioles of patients with resistant hypertension and in mouse arteries (Good et al., 2018a). Moreover, Spironolactone was shown to reduce blood pressure in mice *via* inhibition of Panx1 channels in vascular SMCs (independently of its effect on MRs) (Good et al., 2018a). Like many drugs, Spironolactone was found to bind to plasma proteins in the circulation, which considerably impairs its diffusion into tissues. Furthermore, Spironolactone is almost 1000-times more potent to block MRs than Panx1 channels, which limits its potential application as a Panx1 inhibitory medication (Huke, 2018).

The exact mechanism by which many of the above-mentioned compounds exert their Panx1 inhibitory action remains unknown. It is believed that different Panx1 channel blockers may have different modes of action and they possibly target distinct binding sites. Moreover, some of the molecules can block the Panx1 channel completely by means of steric hindrance while others act on certain sub-conductance states (Dahl et al., 2013). An in-depth characterization of the structural and electrophysiological properties of Panx1 channels remains of great importance for the future development of new pharmacological tools.

## Novel classes of Panx1 channel inhibitors: A potential approach to improve the specificity

Re-purposing of drugs or unbiased searches in small molecule libraries has this far not yet resulted in the finding of a specific Panx1 channel inhibitor (Dahl et al., 2013). Innovative approaches are thus needed. Such means may include the use of synthetic peptides or small antibodies (“mini-antibodies”), which selectively target a given sequence of the Panx1 molecule and potentially lead to more specific effects (Molica et al., 2019; Lian et al., 2021).

Synthetic peptides represent a highly specific class of molecules. They are designed to target a given sequence, thus they provide a useful tool in both fundamental and clinical research (Lian et al., 2021). Several peptides against Panx1 channels have been produced and their inhibitory capacity was confirmed in independent publications (Pelegrin and Surprenant, 2006; Weilinger et al., 2012; Billaud et al., 2015).

As early as in 2006, Pelegrin and Surprenant generated anti-Panx1 peptides that targeted the extracellular domains of Panx1 (Pelegrin and Surprenant, 2006). Among them, ^10^Panx1 with its 10-amino acid sequence (WRQAAFVDSY) mimics residues 74–83 of the Panx1 EL1, of which W74 and R75 are now known to play an essential role in the blocking of the channel (Pelegrin and Surprenant, 2006; Michalski et al., 2020). The ^10^Panx1 peptide showed the potential to specifically block the Panx1 channel and P2X7R-mediated dye uptake but without affecting P2X7R ion current in different cell types e.g., transfected HEK-923 or HeLa cells, mouse J774 macrophages, human lung alveolar macrophages or THP-1 cells (Pelegrin and Surprenant, 2006). The Panx1 ion currents were reversibly inhibited by ^10^Panx1 peptides, albeit to a lesser extent than with Cbx (Pelegrin and Surprenant, 2006). The latter is not surprising given that Cbx is not selective for Panx1 channels. Follow-up studies using ^10^Panx1 explored Panx1 channel function during the process of caspase-1 activation (Brough et al., 2009). It was shown that ^10^Panx1 reduced ATP release and inhibited dye uptake in astrocytes and counteracted P2R stimulation in murine macrophages (Willebrords et al., 2017). In addition to its anti-inflammatory actions, beneficial effects of ^10^Panx1 were shown in Panx1-mediated platelet aggregation (Molica et al., 2019), α-adrenergic vasoconstriction (Billaud et al., 2011), Panx1-related HIV replication (Seror et al., 2011) and breast cancer metastasis in mice (Furlow et al., 2015). Even though the ^10^Panx1 peptide was designed to target the Panx1 EL1, it was also shown to inhibit Cx46 current although to a lesser extent (Pelegrin and Surprenant, 2006; Dahl, 2007; Wang et al., 2007). In addition, some anti-purinergic actions were found, of which the mechanism remains so far unexplained (Pelegrin and Surprenant, 2006; Wang et al., 2007). These potential off-target effects question the specificity of the ^10^Panx1 peptide and call for additional developments (Chiu et al., 2018). Moreover, the low stability of ^10^Panx1 in plasma limits the potential *in vivo* application (Molica et al., 2019).

Phosphorylation of the tyrosine residue at Y198 within the Panx1 IL domain is involved in the channel gating (Good et al., 2018a; DeLalio et al., 2020). A membrane-permeable peptide, called PxIL2P, was developed to target this tyrosine residue. The sequence consists of 10 residues (191-KYPIVEQYLK-200) and contains a specific TAT sequence (YGRKKQRRR), which helps for the entry into cells. Electrophysiological recordings demonstrated that Panx1 channels were inhibited by PxIL2P. Moreover, the peptide blocked Panx1-mediated ATP release, TNFα-induced IL-1β secretion and α-adrenergic vasoconstriction (Billaud et al., 2015; Good et al., 2018a; Yang et al., 2020). Of note, the inhibitory effect of PxIL2P on Phenylephrine-induced vasoconstriction was smaller than the ones evoked by Pbn or ^10^Panx1 (Billaud et al., 2015). Considering the above-mentioned potential off-target effects of Pbn or ^10^Panx1, the lower level of inhibition by PxIL2P may possibly point to a higher specificity of this peptide (Billaud et al., 2015).

Another peptide, namely TAT-Panx_308_, mimics the Y308 phosphorylation site localized in the CT of Panx1. Exposure of cells to this peptide prevented Panx1-Y308 phosphorylation and thereby the opening of the channel (Weilinger et al., 2016; Good et al., 2018a). The peptide contains a characteristic TAT sequence allowing for easy cell penetration. Inhibition of Panx1 channels with TAT-Panx_308_ was shown to confer neuroprotection after middle cerebral artery occlusion (MCAO) in rats and decreased infarct size after stroke (Weilinger et al., 2016).

A caveat of peptide-based blockers appeared in the experimental use of scrambled control peptides, which showed sometimes blocking properties as well (Dahl, 2007). This applies mainly to peptides that base their blocking properties on steric hindrance with the channel pore. In some cases, the folding of the scrambled version can lead to a dimension that also pursues channel inhibition (Wang et al., 2007; Dahl et al., 2013). Although such effects seem at first glance undesirable and indeed call for better controls or a reorganized study design, one may also learn from this finding that steric hindrance alone and approximate similarity is enough to inhibit the Panx1 channel.

**FIGURE 2:**
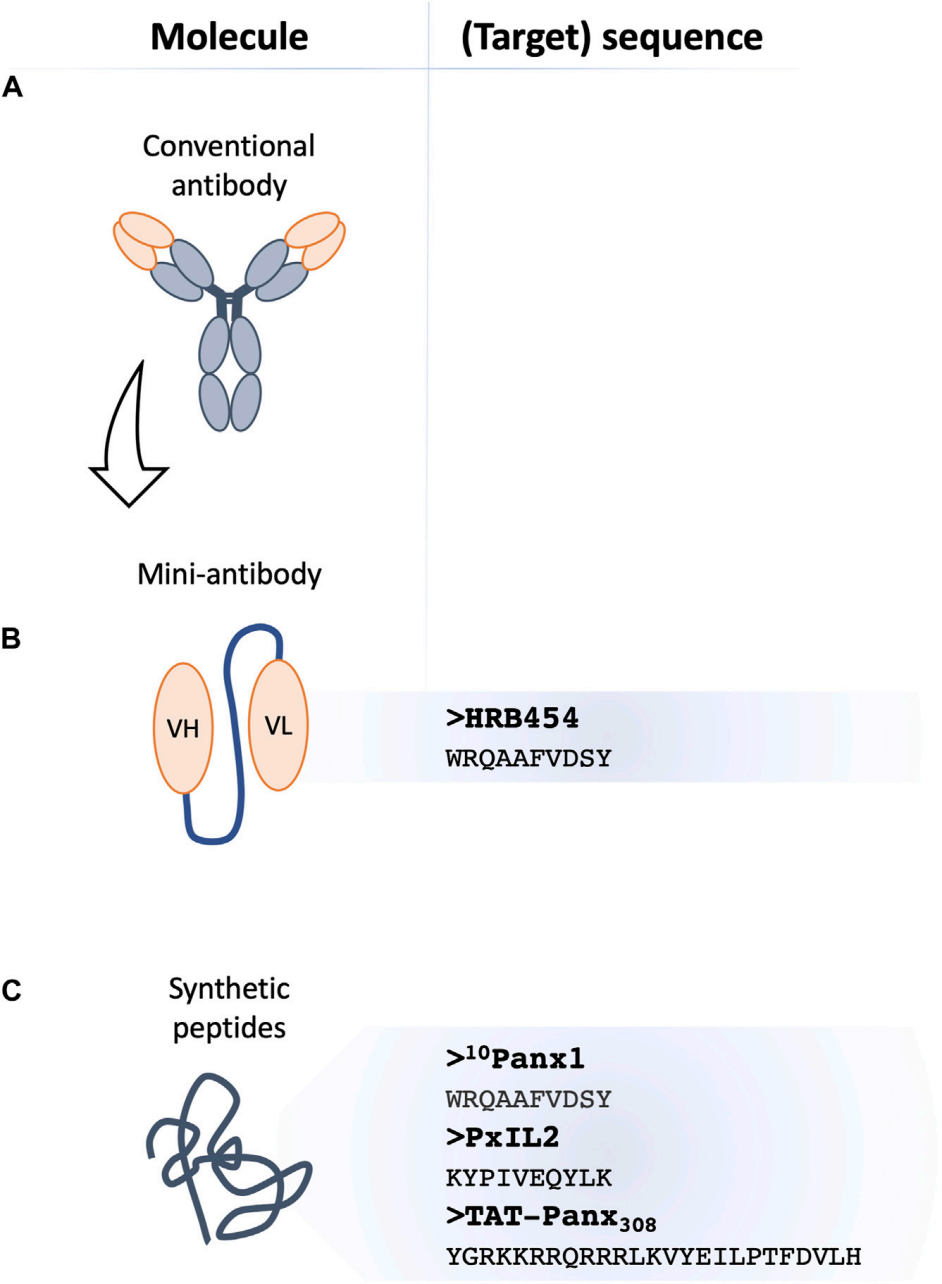
Novel classes of Panx1 channel inhibitors. Membrane channel inhibitors comprise antibody-based strategies, where conventional monoclonal antibodies **(A)** with a channel blocking capacity appear as an attractive tool in biotechnology. The single variable fragment (scFv)-based agents known as ‘mini-antibodies’ exhibit better tissue penetration and fewer adverse effects due to their reduced size. The scFv-based against Panx1 **(B)**, referred to as HRB454, with its target sequence is shown at the right. Mimetic peptides **(C)** have all sequence homology to Panx1 and their blocking capacity can be based on interference with Panx1 phosphorylation by providing an additional substrate to a kinase (e.g., TAT-Panx_308_) or by disturbing (other) protein-protein interactions (e.g., ^10^Panx1 and PxIL2). PxIL2 and TAT-Panx_308_ peptides target intracellular Panx1 domains, thus TAT-conjugation or inclusion in patch pipette solution will allow for optimal efficacy. The published sequences of peptide-based inhibitors are shown on the right.

Monoclonal antibodies (mAbs) have recently been popularized as specific and potent channel inhibitors (Haustrate et al., 2019). mAbs are produced by B cells to specifically recognize given antigens (Lu et al., 2020). In 1975, Köhler and Milstein introduced the hybridoma-based technique allowing for the generation of large amounts of mAbs, which contributed to the development of multiple applications in the biotechnology industry and clinics (Kohler and Milstein, 1975; Lu et al., 2020). Antibody-based channel inhibitors targeting the Panx1-CT were shown to be effective in patch clamp studies when perfused into the cytosol (Weilinger et al., 2012; Weilinger et al., 2016; Bialecki et al., 2020). Progression of innovative technologies to create recombinant mAbs expanded the field and gave rise to the single-chain variable fragments (scFv, further called “mini-antibody”) that consist of the variable regions of the heavy and light chain of immunoglobulin (VH and VL, respectively) connected by a peptide linker (Figure 2) (Ahmad et al., 2012; de Aguiar et al., 2021; Jin et al., 2022). Nowadays’ strategies apply phage display-based libraries that use filamentous bacteriophages expressing the target as a fusion with their coat protein (Ahmad et al., 2012; Wu et al., 2016; Lu et al., 2020). Surface expression of the antigen facilitates the selection of specific binders and affinity studies (Sebollela et al., 2017; Lu et al., 2020). Due to their reduced size, mini-antibodies exhibit better tissue penetration and fewer fragment crystallizable region (Fc)-mediated adverse effects, while still exhibiting the same affinity to the target as a full-length antibody (Jin et al., 2022). Recombinant mAbs are currently approved as a therapeutic agent for a wide range of targets in immunology (Lu et al., 2020), oncology (Davda et al., 2019; Jin et al., 2022), cardiovascular diseases (Lu et al., 2020) and many other applications (Lu et al., 2020). Several scFv’s have been recognized as blocking molecules against their targets (Yi et al., 2001; Ahmad et al., 2012; Sebollela et al., 2017).

With the help of the Geneva Antibody Facility (https://www.unige.ch/medecine/antibodies/), our group has recently raised a panel of mini-antibodies against a sequence (WRQAAFVDSY) in the EL1 of Panx1 (Molica et al., 2019; Rusiecka et al., 2019). The antigen-binding fragment was fused to the human Fc (Ahmad et al., 2012; Molica et al., 2019). While some of the mini-antibodies appeared very useful for immunofluorescent microscopy, the anti-Panx1 HRB454 appeared to hold Panx1 channel inhibiting properties (Molica et al., 2019; Rusiecka et al., 2019). In *in vivo* mouse models of venous and arterial thrombosis, HRB454 decreased hemostasis and thrombosis *via* inhibition of Panx1 channels (Molica et al., 2019). This study promotes further research into mini-antibody-based inhibition of Panx1 channels for diseases involving inflammation or cell death. Potential side effects of long-term application of HRB454 as well as off-target effects remain to be investigated. In general, the efficacy of tissue penetration and retention can constitute a limiting factor for application of mini-antibodies and also needs to be examined for HRB454. Moreover, the Fc region prolongs the half-life of mini-antibodies, yet it may also interact with distinct receptors present on the surface of different cell types, affecting the molecules’ retention (Chames et al., 2009). Moreover, the fusion with the Fc increases the size of the molecule, which may be deleterious for the penetration to less accessible targets. Although one would expect relative long plasma stability of anti-Panx1 mini-antibodies, this remains to be confirmed (Chames et al., 2009; Molica et al., 2019).

## Conclusion

Panx1 is a ubiquitously expressed protein that mediates paracrine and autocrine signaling and regulates a plethora of physiological and pathological processes. Panx1 channels have been shown to regulate the inflammatory response by various mechanisms, and the role of this channel as modifier of immune and inflammatory pathologies is emerging. Pharmacological inhibition of Panx1 channels has been proven beneficial *in vivo*, i.e. it reduces the inflammatory response and mitigates severe disease outcomes associated with inflammation in different organs. Unfortunately, currently available Panx1 blockers lack specificity and/or *in vivo* stability. Next to several off-target effects, many of them induce unwanted clinical side effects that further limit their application. Innovative approaches such as synthetic peptides and scFv-based inhibitors give promising results and may provide in the future a means to selectively reduce Panx1 channel activity for therapeutic purposes.

## References

[B1] Adrover J. M. Del Fresno C. Crainiciuc G. Cuartero M. I. Casanova-Acebes M. Weiss L. A. (2019). A neutrophil timer coordinates immune defense and vascular protection. Immunity 50 (2), 390–402. e310. 10.1016/j.immuni.2019.01.002 30709741

[B2] Ahmad Z. A. Yeap S. K. Ali A. M. Ho W. Y. Alitheen N. B. Hamid M. (2012). scFv antibody: principles and clinical application. Clin. Dev. Immunol. 2012, 980250. 10.1155/2012/980250 22474489 PMC3312285

[B3] Bao L. Locovei S. Dahl G. (2004). Pannexin membrane channels are mechanosensitive conduits for ATP. FEBS Lett. 572 (1-3), 65–68. 10.1016/j.febslet.2004.07.009 15304325

[B4] Bao Y. Chen Y. Ledderose C. Li L. Junger W. G. (2013). Pannexin 1 channels link chemoattractant receptor signaling to local excitation and global inhibition responses at the front and back of polarized neutrophils. J. Biol. Chem. 288 (31), 22650–22657. 10.1074/jbc.M113.476283 23798685 PMC3829350

[B5] Bialecki J. Werner A. Weilinger N. L. Tucker C. M. Vecchiarelli H. A. Egana J. (2020). Suppression of presynaptic glutamate release by postsynaptic metabotropic NMDA receptor signalling to pannexin-1. J. Neurosci. 40 (4), 729–742. 10.1523/JNEUROSCI.0257-19.2019 31818976 PMC6975291

[B6] Billaud M. Chiu Y. H. Lohman A. W. Parpaite T. Butcher J. T. Mutchler S. M. (2015). A molecular signature in the pannexin1 intracellular loop confers channel activation by the α1 adrenoreceptor in smooth muscle cells. Sci. Signal. 8 (364), ra17. 10.1126/scisignal.2005824 25690012 PMC4358815

[B7] Billaud M. Lohman A. W. Straub A. C. Looft-Wilson R. Johnstone S. R. Araj C. A. (2011). Pannexin1 regulates α1-adrenergic receptor- mediated vasoconstriction. Circ. Res. 109 (1), 80–85. 10.1161/CIRCRESAHA.110.237594 21546608 PMC3135971

[B8] Boassa D. Ambrosi C. Qiu F. Dahl G. Gaietta G. Sosinsky G. (2007). Pannexin1 channels contain a glycosylation site that targets the hexamer to the plasma membrane. J. Biol. Chem. 282 (43), 31733–31743. 10.1074/jbc.M702422200 17715132

[B9] Boassa D. Qiu F. Dahl G. Sosinsky G. (2008). Trafficking dynamics of glycosylated pannexin 1 proteins. Cell Commun. Adhes. 15 (1), 119–132. 10.1080/15419060802013885 18649184 PMC2528835

[B10] Boyce A. K. J. Epp A. L. Nagarajan A. Swayne L. A. (2018). Transcriptional and post-translational regulation of pannexins. Biochim. Biophys. Acta. Biomembr. 1860 (1), 72–82. 10.1016/j.bbamem.2017.03.004 28279657

[B11] Brough D. Pelegrin P. Rothwell N. J. (2009). Pannexin-1-dependent caspase-1 activation and secretion of IL-1beta is regulated by zinc. Eur. J. Immunol. 39 (2), 352–358. 10.1002/eji.200838843 19130485 PMC2696683

[B12] Bruzzone R. Barbe M. T. Jakob N. J. Monyer H. (2005). Pharmacological properties of homomeric and heteromeric pannexin hemichannels expressed in Xenopus oocytes. J. Neurochem. 92 (5), 1033–1043. 10.1111/j.1471-4159.2004.02947.x 15715654

[B13] Celetti S. J. Cowan K. N. Penuela S. Shao Q. Churko J. Laird D. W. (2010). Implications of pannexin 1 and pannexin 3 for keratinocyte differentiation. J. Cell Sci. 123 (8), 1363–1372. 10.1242/jcs.056093 20332104

[B14] Chames P. Van Regenmortel M. Weiss E. Baty D. (2009). Therapeutic antibodies: Successes, limitations and hopes for the future. Br. J. Pharmacol. 157 (2), 220–233. 10.1111/j.1476-5381.2009.00190.x 19459844 PMC2697811

[B15] Chekeni F. B. Elliott M. R. Sandilos J. K. Walk S. F. Kinchen J. M. Lazarowski E. R. (2010). Pannexin 1 channels mediate 'find-me' signal release and membrane permeability during apoptosis. Nature 467 (7317), 863–867. 10.1038/nature09413 20944749 PMC3006164

[B16] Chen K. W. Demarco B. Broz P. (2020). Pannexin-1 promotes NLRP3 activation during apoptosis but is dispensable for canonical or noncanonical inflammasome activation. Eur. J. Immunol. 50 (2), 170–177. 10.1002/eji.201948254 31411729

[B17] Chen Y. Corriden R. Inoue Y. Yip L. Hashiguchi N. Zinkernagel A. (2006). ATP release guides neutrophil chemotaxis via P2Y2 and A3 receptors. Science 314 (5806), 1792–1795. 10.1126/science.1132559 17170310

[B18] Chiu Y. H. Ravichandran K. S. Bayliss D. A. (2014). Intrinsic properties and regulation of Pannexin 1 channel. Channels (Austin) 8 (2), 103–109. 10.4161/chan.27545 24419036 PMC4048298

[B19] Chiu Y. H. Schappe M. S. Desai B. N. Bayliss D. A. (2018). Revisiting multimodal activation and channel properties of Pannexin 1. J. Gen. Physiol. 150 (1), 19–39. 10.1085/jgp.201711888 29233884 PMC5749114

[B20] Chu K. Yin B. Wang J. Peng G. Liang H. Xu Z. (2012). Inhibition of P2X7 receptor ameliorates transient global cerebral ischemia/reperfusion injury via modulating inflammatory responses in the rat hippocampus. J. Neuroinflammation 9, 69. 10.1186/1742-2094-9-69 22513224 PMC3418181

[B21] Cisneros-Mejorado A. Gottlieb M. Cavaliere F. Magnus T. Koch-Nolte F. Scemes E. (2015). Blockade of P2X7 receptors or pannexin-1 channels similarly attenuates postischemic damage. J. Cereb. Blood Flow. Metab. 35 (5), 843–850. 10.1038/jcbfm.2014.262 25605289 PMC4420860

[B22] Crespo Yanguas S. Willebrords J. Johnstone S. R. Maes M. Decrock E. De Bock M. (2017). Pannexin1 as mediator of inflammation and cell death. Biochim. Biophys. Acta. Mol. Cell Res. 1864 (1), 51–61. 10.1016/j.bbamcr.2016.10.006 27741412 PMC5693326

[B23] Cruikshank S. J. Hopperstad M. Younger M. Connors B. W. Spray D. C. Srinivas M. (2004). Potent block of Cx36 and Cx50 gap junction channels by mefloquine. Proc. Natl. Acad. Sci. U. S. A. 101 (33), 12364–12369. 10.1073/pnas.0402044101 15297615 PMC514481

[B24] D'Hondt C. Ponsaerts R. De Smedt H. Vinken M. De Vuyst E. De Bock M. (2011). Pannexin channels in ATP release and beyond: An unexpected rendezvous at the endoplasmic reticulum. Cell. Signal. 23 (2), 305–316. 10.1016/j.cellsig.2010.07.018 20688156

[B25] Dahl G. (2007). Gap junction-mimetic peptides do work, but in unexpected ways. Cell Commun. Adhes. 14 (6), 259–264. 10.1080/15419060801891018 18392993

[B26] Dahl G. Keane R. W. (2012). Pannexin: From discovery to bedside in 11+/-4 years? Brain Res. 1487, 150–159. 10.1016/j.brainres.2012.04.058 22771709 PMC3590907

[B27] Dahl G. Qiu F. Wang J. (2013). The bizarre pharmacology of the ATP release channel pannexin1. Neuropharmacology 75, 583–593. 10.1016/j.neuropharm.2013.02.019 23499662 PMC3711969

[B28] Davda J. Declerck P. Hu-Lieskovan S. Hickling T. P. Jacobs I. A. Chou J. (2019). Immunogenicity of immunomodulatory, antibody-based, oncology therapeutics. J. Immunother. Cancer 7 (1), 105. 10.1186/s40425-019-0586-0 30992085 PMC6466770

[B29] de Aguiar R. B. de Almeida da Silva T. Costa B. A. Machado M. F. M. Yamada R. Y. Braggion C. (2021). Generation and functional characterization of a single-chain variable fragment (scFv) of the anti-FGF2 3F12E7 monoclonal antibody. Sci. Rep. 11 (1), 1432. 10.1038/s41598-020-80746-8 33446839 PMC7809466

[B30] DeLalio L. J. Keller A. S. Chen J. Boyce A. K. J. Artamonov M. V. Askew-Page H. R. (2018). Interaction between pannexin 1 and caveolin-1 in smooth muscle can regulate blood pressure. Arterioscler. Thromb. Vasc. Biol. 38 (9), 2065–2078. 10.1161/ATVBAHA.118.311290 30026274 PMC6202122

[B31] DeLalio L. J. Masati E. Mendu S. Ruddiman C. A. Yang Y. Johnstone S. R. (2020). Pannexin 1 channels in renin-expressing cells influence renin secretion and blood pressure homeostasis. Kidney Int. 98 (3), 630–644. 10.1016/j.kint.2020.04.041 32446934 PMC7483468

[B32] Delmar M. Laird D. W. Naus C. C. Nielsen M. S. Verselis V. K. White T. W. (2018). Connexins and disease. Cold Spring Harb. Perspect. Biol. 10 (9), a029348. 10.1101/cshperspect.a029348 28778872 PMC6120696

[B33] Deng Z. He Z. Maksaev G. Bitter R. M. Rau M. Fitzpatrick J. A. J. (2020). Cryo-EM structures of the ATP release channel pannexin 1. Nat. Struct. Mol. Biol. 27 (4), 373–381. 10.1038/s41594-020-0401-0 32231289

[B34] Dolmatova E. Spagnol G. Boassa D. Baum J. R. Keith K. Ambrosi C. (2012). Cardiomyocyte ATP release through pannexin 1 aids in early fibroblast activation. Am. J. Physiol. Heart Circ. Physiol. 303 (10), H1208–H1218. 10.1152/ajpheart.00251.2012 22982782 PMC3517637

[B35] Douanne T. Andre-Gregoire G. Trillet K. Thys A. Papin A. Feyeux M. (2019). Pannexin-1 limits the production of proinflammatory cytokines during necroptosis. EMBO Rep. 20 (10), e47840. 10.15252/embr.201947840 31410978 PMC6776911

[B36] Dubyak G. R. (2002). Focus on "extracellular ATP signaling and P2X nucleotide receptors in monolayers of primary human vascular endothelial cells. Am. J. Physiol. Cell Physiol. 282 (2), C242–C244. 10.1152/ajpcell.00522.2001 11788334

[B37] Elmore S. (2007). Apoptosis: A review of programmed cell death. Toxicol. Pathol. 35 (4), 495–516. 10.1080/01926230701320337 17562483 PMC2117903

[B38] Ferreira L. G. Faria R. X. Ferreira N. C. Soares-Bezerra R. J. (2016). Brilliant Blue dyes in daily food: How could purinergic system Be affected? Int. J. Food Sci. 2016, 7548498. 10.1155/2016/7548498 27833914 PMC5090090

[B39] Fotis L. Agrogiannis G. Vlachos I. S. Pantopoulou A. Margoni A. Kostaki M. (2012). Intercellular adhesion molecule (ICAM)-1 and vascular cell adhesion molecule (VCAM)-1 at the early stages of atherosclerosis in a rat model. Vivo 26 (2), 243–250. 22351665

[B40] Freeman T. J. Sayedyahossein S. Johnston D. Sanchez-Pupo R. E. O'Donnell B. Huang K. (2019). Inhibition of pannexin 1 reduces the tumorigenic properties of human melanoma cells. Cancers (Basel) 11 (1), 102. 10.3390/cancers11010102 30654593 PMC6356688

[B41] Furlow P. W. Zhang S. Soong T. D. Halberg N. Goodarzi H. Mangrum C. (2015). Mechanosensitive pannexin-1 channels mediate microvascular metastatic cell survival. Nat. Cell Biol. 17 (7), 943–952. 10.1038/ncb3194 26098574 PMC5310712

[B42] Gaynullina D. Shestopalov V. I. Panchin Y. Tarasova O. S. (2015). Pannexin 1 facilitates arterial relaxation via an endothelium-derived hyperpolarization mechanism. FEBS Lett. 589 (10), 1164–1170. 10.1016/j.febslet.2015.03.018 25819435

[B43] Gehi R. Shao Q. Laird D. W. (2011). Pathways regulating the trafficking and turnover of pannexin1 protein and the role of the C-terminal domain. J. Biol. Chem. 286 (31), 27639–27653. 10.1074/jbc.M111.260711 21659516 PMC3149355

[B44] Geissmann F. Manz M. G. Jung S. Sieweke M. H. Merad M. Ley K. (2010). Development of monocytes, macrophages, and dendritic cells. Science 327 (5966), 656–661. 10.1126/science.1178331 20133564 PMC2887389

[B45] Good M. E. Chiu Y. H. Poon I. K. H. Medina C. B. Butcher J. T. Mendu S. K. (2018a). Pannexin 1 channels as an unexpected new target of the anti-hypertensive drug Spironolactone. Circ. Res. 122 (4), 606–615. 10.1161/CIRCRESAHA.117.312380 29237722 PMC5815904

[B46] Good M. E. Eucker S. A. Li J. Bacon H. M. Lang S. M. Butcher J. T. (2018b). Endothelial cell Pannexin1 modulates severity of ischemic stroke by regulating cerebral inflammation and myogenic tone. JCI Insight 3 (6), e96272. 10.1172/jci.insight.96272 29563335 PMC5926909

[B47] Good M. E. Young A. P. Wolpe A. G. Ma M. Hall P. J. Duffy C. K. (2021). Endothelial pannexin 1 regulates cardiac response to myocardial infarction. Circ. Res. 128 (8), 1211–1213. 10.1161/CIRCRESAHA.120.317272 33641341 PMC8049979

[B48] Haustrate A. Hantute-Ghesquier A. Prevarskaya N. Lehen'kyi V. (2019). Monoclonal antibodies targeting ion channels and their therapeutic potential. Front. Pharmacol. 10, 606. 10.3389/fphar.2019.00606 31231216 PMC6561378

[B49] Herve J. C. Derangeon M. (2013). Gap-junction-mediated cell-to-cell communication. Cell Tissue Res. 352 (1), 21–31. 10.1007/s00441-012-1485-6 22940728

[B50] Huang Y. Lin J. Chen X. Lin J. (2021). Pannexin-1 contributes to the apoptosis of spinal neurocytes in spinal cord injury. Front. Physiol. 12, 656647. 10.3389/fphys.2021.656647 33986693 PMC8112589

[B51] Huke S. (2018). Pannexin channel inhibition: An evolving target to lower blood pressure? Circ. Res. 122 (4), 543–545. 10.1161/CIRCRESAHA.118.312566 29449359 PMC5821142

[B52] Iglesias R. Locovei S. Roque A. Alberto A. P. Dahl G. Spray D. C. (2008). P2X7 receptor-pannexin1 complex: Pharmacology and signaling. Am. J. Physiol. Cell Physiol. 295 (3), C752–C760. 10.1152/ajpcell.00228.2008 18596211 PMC2544446

[B53] Iglesias R. Spray D. C. Scemes E. (2009). Mefloquine blockade of Pannexin1 currents: Resolution of a conflict. Cell Commun. Adhes. 16 (5-6), 131–137. 10.3109/15419061003642618 20218915 PMC2854254

[B54] Jankowski J. Perry H. M. Medina C. B. Huang L. Yao J. Bajwa A. (2018). Epithelial and endothelial Pannexin1 channels mediate AKI. J. Am. Soc. Nephrol. 29 (7), 1887–1899. 10.1681/ASN.2017121306 29866797 PMC6050932

[B55] Jiang J. X. Penuela S. (2016). Connexin and pannexin channels in cancer. BMC Cell Biol. 17 (1), 12. 10.1186/s12860-016-0094-8 27229305 PMC4896247

[B56] Jin Q. Zhang B. Zheng X. Li N. Xu L. Xie Y. (2020). Cryo-EM structures of human pannexin 1 channel. Cell Res. 30 (5), 449–451. 10.1038/s41422-020-0310-0 32246089 PMC7196119

[B57] Jin S. Sun Y. Liang X. Gu X. Ning J. Xu Y. (2022). Emerging new therapeutic antibody derivatives for cancer treatment. Signal Transduct. Target. Ther. 7 (1), 39. 10.1038/s41392-021-00868-x 35132063 PMC8821599

[B58] Kawamura M. Jr. Ruskin D. N. Masino S. A. (2010). Metabolic autocrine regulation of neurons involves cooperation among pannexin hemichannels, adenosine receptors, and KATP channels. J. Neurosci. 30 (11), 3886–3895. 10.1523/JNEUROSCI.0055-10.2010 20237259 PMC2872120

[B59] Kirby B. S. Sparks M. A. Lazarowski E. R. Lopez Domowicz D. A. Zhu H. McMahon T. J. (2021). Pannexin 1 channels control the hemodynamic response to hypoxia by regulating O2-sensitive extracellular ATP in blood. Am. J. Physiol. Heart Circ. Physiol. 320 (3), H1055–H1065. 10.1152/ajpheart.00651.2020 33449849 PMC7988759

[B60] Kohler G. Milstein C. (1975). Continuous cultures of fused cells secreting antibody of predefined specificity. Nature 256 (5517), 495–497. 10.1038/256495a0 1172191

[B61] Koval M. Cwiek A. Carr T. Good M. E. Lohman A. W. Isakson B. E. (2021). Pannexin 1 as a driver of inflammation and ischemia-reperfusion injury. Purinergic Signal. 17 (4), 521–531. 10.1007/s11302-021-09804-8 34251590 PMC8273370

[B62] Kumar V. A. A. K. Aster J. C. (2017). “Robbins basic pathology,” in Robbins basic pathology. 10 ed (Elsevier - Health Sciences Division), 57–96.

[B63] Kuzuya M. Hirano H. Hayashida K. Watanabe M. Kobayashi K. Terada T. (2022). Structures of human pannexin-1 in nanodiscs reveal gating mediated by dynamic movement of the N terminus and phospholipids. Sci. Signal. 15 (720), eabg6941. 10.1126/scisignal.abg6941 35133866

[B64] Le Vasseur M. Lelowski J. Bechberger J. F. Sin W. C. Naus C. C. (2014). Pannexin 2 protein expression is not restricted to the CNS. Front. Cell. Neurosci. 8, 392. 10.3389/fncel.2014.00392 25505382 PMC4243559

[B65] Lee N. S. Yoon C. W. Wang Q. Moon S. Koo K. M. Jung H. (2020). Focused ultrasound stimulates ER localized mechanosensitive PANNEXIN-1 to mediate intracellular calcium release in invasive cancer cells. Front. Cell Dev. Biol. 8, 504. 10.3389/fcell.2020.00504 32656213 PMC7325310

[B66] Lee V. R. Barr K. J. Kelly J. J. Johnston D. Brown C. F. C. Robb K. P. (2018). Pannexin 1 regulates adipose stromal cell differentiation and fat accumulation. Sci. Rep. 8 (1), 16166. 10.1038/s41598-018-34234-9 30385873 PMC6212408

[B67] Lemaire I. Falzoni S. Zhang B. Pellegatti P. Di Virgilio F. (2011). The P2X7 receptor and Pannexin-1 are both required for the promotion of multinucleated macrophages by the inflammatory cytokine GM-CSF. J. Immunol. 187 (7), 3878–3887. 10.4049/jimmunol.1002780 21865551

[B68] Ley K. (2003). Healing without inflammation? Am. J. Physiol. Regul. Integr. Comp. Physiol. 285 (4), R718–R719. 10.1152/ajpregu.00318.2003 12959915

[B69] Ley K. Laudanna C. Cybulsky M. I. Nourshargh S. (2007). Getting to the site of inflammation: The leukocyte adhesion cascade updated. Nat. Rev. Immunol. 7 (9), 678–689. 10.1038/nri2156 17717539

[B70] Lian Z. Wang N. Tian Y. Huang L. (2021). Characterization of synthetic peptide therapeutics using liquid chromatography-mass spectrometry: Challenges, solutions, pitfalls, and future perspectives. J. Am. Soc. Mass Spectrom. 32 (8), 1852–1860. 10.1021/jasms.0c00479 34110145

[B71] Lillo M. A. Gaete P. S. Puebla M. Burboa P. C. Poblete I. Figueroa X. F. (2021). Novel pannexin-1-coupled signaling cascade involved in the control of endothelial cell function and NO-dependent relaxation. Oxid. Med. Cell. Longev. 2021, 2678134. 10.1155/2021/2678134 33688389 PMC7914086

[B72] Lohman A. W. Leskov I. L. Butcher J. T. Johnstone S. R. Stokes T. A. Begandt D. (2015). Pannexin 1 channels regulate leukocyte emigration through the venous endothelium during acute inflammation. Nat. Commun. 6, 7965. 10.1038/ncomms8965 26242575 PMC4824045

[B73] Lopez X. Palacios-Prado N. Guiza J. Escamilla R. Fernandez P. Vega J. L. (2021). A physiologic rise in cytoplasmic calcium ion signal increases pannexin1 channel activity via a C-terminus phosphorylation by CaMKII. Proc. Natl. Acad. Sci. U. S. A. 118 (32), e2108967118. 10.1073/pnas.2108967118 34301850 PMC8364136

[B74] Lu R. M. Hwang Y. C. Liu I. J. Lee C. C. Tsai H. Z. Li H. J. (2020). Development of therapeutic antibodies for the treatment of diseases. J. Biomed. Sci. 27 (1), 1. 10.1186/s12929-019-0592-z 31894001 PMC6939334

[B75] Ma W. Compan V. Zheng W. Martin E. North R. A. Verkhratsky A. (2012). Pannexin 1 forms an anion-selective channel. Pflugers Arch. 463 (4), 585–592. 10.1007/s00424-012-1077-z 22311122

[B76] Ma W. Hui H. Pelegrin P. Surprenant A. (2009). Pharmacological characterization of pannexin-1 currents expressed in mammalian cells. J. Pharmacol. Exp. Ther. 328 (2), 409–418. 10.1124/jpet.108.146365 19023039 PMC2682283

[B77] Maier-Begandt D. Comstra H. S. Molina S. A. Kruger N. Ruddiman C. A. Chen Y. L. (2021). A venous-specific purinergic signaling cascade initiated by Pannexin 1 regulates TNFα-induced increases in endothelial permeability. Sci. Signal. 14 (672), eaba2940. 10.1126/scisignal.aba2940 33653920 PMC8011850

[B78] Makarenkova H. P. Shah S. B. Shestopalov V. I. (2018). The two faces of pannexins: New roles in inflammation and repair. J. Inflamm. Res. 11, 273–288. 10.2147/JIR.S128401 29950881 PMC6016592

[B79] Makarenkova H. P. Shestopalov V. I. (2014). The role of pannexin hemichannels in inflammation and regeneration. Front. Physiol. 5, 63. 10.3389/fphys.2014.00063 24616702 PMC3933922

[B80] Margraf A. Ley K. Zarbock A. (2019). Neutrophil recruitment: From model systems to tissue-specific patterns. Trends Immunol. 40 (7), 613–634. 10.1016/j.it.2019.04.010 31175062 PMC6745447

[B81] McNally A. K. Anderson J. M. (2011). Macrophage fusion and multinucleated giant cells of inflammation. Adv. Exp. Med. Biol. 713, 97–111. 10.1007/978-94-007-0763-4_7 21432016

[B82] McNally A. K. Anderson J. M. (2005). Multinucleated giant cell formation exhibits features of phagocytosis with participation of the endoplasmic reticulum. Exp. Mol. Pathol. 79 (2), 126–135. 10.1016/j.yexmp.2005.06.008 16109404

[B83] Medina C. B. Ravichandran K. S. (2016). Do not let death do us part: 'find-me' signals in communication between dying cells and the phagocytes. Cell Death Differ. 23 (6), 979–989. 10.1038/cdd.2016.13 26891690 PMC4987731

[B84] Michalski K. Kawate T. (2016). Carbenoxolone inhibits Pannexin1 channels through interactions in the first extracellular loop. J. Gen. Physiol. 147 (2), 165–174. 10.1085/jgp.201511505 26755773 PMC4727946

[B85] Michalski K. Syrjanen J. L. Henze E. Kumpf J. Furukawa H. Kawate T. (2020). The Cryo-EM structure of pannexin 1 reveals unique motifs for ion selection and inhibition. Elife 9, e54670. 10.7554/eLife.54670 32048993 PMC7108861

[B86] Molica F. Figueroa X. F. Kwak B. R. Isakson B. E. Gibbins J. M. (2018). Connexins and pannexins in vascular function and disease. Int. J. Mol. Sci. 19 (6), 1663. 10.3390/ijms19061663 29874791 PMC6032213

[B87] Molica F. Meens M. J. Pelli G. Hautefort A. Emre Y. Imhof B. A. (2019). Selective inhibition of Panx1 channels decreases hemostasis and thrombosis *in vivo* . Thromb. Res. 183, 56–62. 10.1016/j.thromres.2019.09.028 31669824

[B88] Molica F. Morel S. Meens M. J. Denis J. F. Bradfield P. F. Penuela S. (2015). Functional role of a polymorphism in the Pannexin1 gene in collagen-induced platelet aggregation. Thromb. Haemost. 114 (2), 325–336. 10.1160/TH14-11-0981 25947940

[B89] Narahari A. K. Kreutzberger A. J. Gaete P. S. Chiu Y. H. Leonhardt S. A. Medina C. B. (2021). ATP and large signaling metabolites flux through caspase-activated Pannexin 1 channels. Elife 10, e64787. 10.7554/eLife.64787 33410749 PMC7806264

[B90] Navis K. E. Fan C. Y. Trang T. Thompson R. J. Derksen D. J. (2020). Pannexin 1 channels as a therapeutic target: Structure, inhibition, and outlook. ACS Chem. Neurosci. 11 (15), 2163–2172. 10.1021/acschemneuro.0c00333 32639715

[B91] Palacios-Prado N. Soto P. A. Lopez X. Choi E. J. Marquez-Miranda V. Rojas M. (2022). Endogenous pannexin1 channels form functional intercellular cell-cell channels with characteristic voltage-dependent properties. Proc. Natl. Acad. Sci. U. S. A. 119 (18), e2202104119. 10.1073/pnas.2202104119 35486697 PMC9171361

[B92] Panchin Y. Kelmanson I. Matz M. Lukyanov K. Usman N. Lukyanov S. (2000). A ubiquitous family of putative gap junction molecules. Curr. Biol. 10 (13), R473–R474. 10.1016/s0960-9822(00)00576-5 10898987

[B93] Pelegrin P. Surprenant A. (2006). Pannexin-1 mediates large pore formation and interleukin-1beta release by the ATP-gated P2X7 receptor. EMBO J. 25 (21), 5071–5082. 10.1038/sj.emboj.7601378 17036048 PMC1630421

[B94] Penuela S. Bhalla R. Nag K. Laird D. W. (2009). Glycosylation regulates pannexin intermixing and cellular localization. Mol. Biol. Cell 20 (20), 4313–4323. 10.1091/mbc.E09-01-0067 19692571 PMC2762227

[B95] Penuela S. Celetti S. J. Bhalla R. Shao Q. Laird D. W. (2008). Diverse subcellular distribution profiles of pannexin 1 and pannexin 3. Cell Commun. Adhes. 15 (1), 133–142. 10.1080/15419060802014115 18649185

[B96] Penuela S. Gehi R. Laird D. W. (2013). The biochemistry and function of pannexin channels. Biochim. Biophys. Acta 1828 (1), 15–22. 10.1016/j.bbamem.2012.01.017 22305965

[B97] Qiu F. Dahl G. (2009). A permeant regulating its permeation pore: Inhibition of pannexin 1 channels by ATP. Am. J. Physiol. Cell Physiol. 296 (2), C250–C255. 10.1152/ajpcell.00433.2008 18945939 PMC2643853

[B98] Qu R. Dong L. Zhang J. Yu X. Wang L. Zhu S. (2020). Cryo-EM structure of human heptameric Pannexin 1 channel. Cell Res. 30 (5), 446–448. 10.1038/s41422-020-0298-5 32203128 PMC7196123

[B99] Qu Y. Misaghi S. Newton K. Gilmour L. L. Louie S. Cupp J. E. (2011). Pannexin-1 is required for ATP release during apoptosis but not for inflammasome activation. J. Immunol. 186 (11), 6553–6561. 10.4049/jimmunol.1100478 21508259

[B100] Riquelme M. A. Cea L. A. Vega J. L. Boric M. P. Monyer H. Bennett M. V. (2013). The ATP required for potentiation of skeletal muscle contraction is released via pannexin hemichannels. Neuropharmacology 75, 594–603. 10.1016/j.neuropharm.2013.03.022 23583931

[B101] Romanov R. A. Bystrova M. F. Rogachevskaya O. A. Sadovnikov V. B. Shestopalov V. I. Kolesnikov S. S. (2012). The ATP permeability of pannexin 1 channels in a heterologous system and in mammalian taste cells is dispensable. J. Cell Sci. 125 (22), 5514–5523. 10.1242/jcs.111062 22956545 PMC3561859

[B102] Rusiecka O. M. Roth C. L. Kwak B. R. Molica F. (2019). RB459 and RB462 antibodies recognize mouse Pannexin1 protein by immunofluorescent staining. Antib. Rep. 2 (2), 39. 10.24450/journals/abrep.2019.e39

[B103] Sandilos J. K. Bayliss D. A. (2012). Physiological mechanisms for the modulation of pannexin 1 channel activity. J. Physiol. 590 (24), 6257–6266. 10.1113/jphysiol.2012.240911 23070703 PMC3533187

[B104] Sandilos J. K. Chiu Y. H. Chekeni F. B. Armstrong A. J. Walk S. F. Ravichandran K. S. (2012). Pannexin 1, an ATP release channel, is activated by caspase cleavage of its pore-associated C-terminal autoinhibitory region. J. Biol. Chem. 287 (14), 11303–11311. 10.1074/jbc.M111.323378 22311983 PMC3322839

[B105] Schenk U. Westendorf A. M. Radaelli E. Casati A. Ferro M. Fumagalli M. (2008). Purinergic control of T cell activation by ATP released through pannexin-1 hemichannels. Sci. Signal. 1 (39), ra6. 10.1126/scisignal.1160583 18827222

[B106] Sebollela A. Cline E. N. Popova I. Luo K. Sun X. Ahn J. (2017). A human scFv antibody that targets and neutralizes high molecular weight pathogenic amyloid-beta oligomers. J. Neurochem. 142 (6), 934–947. 10.1111/jnc.14118 28670737 PMC5752625

[B107] Seo J. H. Dalal M. S. Contreras J. E. (2021). Pannexin-1 channels as mediators of neuroinflammation. Int. J. Mol. Sci. 22 (10), 5189. 10.3390/ijms22105189 34068881 PMC8156193

[B108] Seror C. Melki M. T. Subra F. Raza S. Q. Bras M. Saidi H. (2011). Extracellular ATP acts on P2Y2 purinergic receptors to facilitate HIV-1 infection. J. Exp. Med. 208 (9), 1823–1834. 10.1084/jem.20101805 21859844 PMC3171090

[B109] Sharma A. K. Charles E. J. Zhao Y. Narahari A. K. Baderdinni P. K. Good M. E. (2018). Pannexin-1 channels on endothelial cells mediate vascular inflammation during lung ischemia-reperfusion injury. Am. J. Physiol. Lung Cell. Mol. Physiol. 315 (2), L301-L312–L312. 10.1152/ajplung.00004.2018 29745255 PMC6139659

[B110] Shestopalov V. I. Panchin Y. (2008). Pannexins and gap junction protein diversity. Cell. Mol. Life Sci. 65 (3), 376–394. 10.1007/s00018-007-7200-1 17982731 PMC11131650

[B111] Sica D. A. (2015). Mineralocorticoid receptor antagonists for treatment of hypertension and heart failure. Methodist Debakey cardiovasc. J. 11 (4), 235–239. 10.14797/mdcj-11-4-235 27057293 PMC4814010

[B112] Silverman W. Locovei S. Dahl G. (2008). Probenecid, a gout remedy, inhibits pannexin 1 channels. Am. J. Physiol. Cell Physiol. 295 (3), C761–C767. 10.1152/ajpcell.00227.2008 18596212 PMC2544448

[B113] Silverman W. R. de Rivero Vaccari J. P. Locovei S. Qiu F. Carlsson S. K. Scemes E. (2009). The pannexin 1 channel activates the inflammasome in neurons and astrocytes. J. Biol. Chem. 284 (27), 18143–18151. 10.1074/jbc.M109.004804 19416975 PMC2709345

[B114] Soehnlein O. Steffens S. Hidalgo A. Weber C. (2017). Neutrophils as protagonists and targets in chronic inflammation. Nat. Rev. Immunol. 17 (4), 248–261. 10.1038/nri.2017.10 28287106

[B115] Sosinsky G. E. Boassa D. Dermietzel R. Duffy H. S. Laird D. W. MacVicar B. (2011). Pannexin channels are not gap junction hemichannels. Channels (Austin) 5 (3), 193–197. 10.4161/chan.5.3.15765 21532340 PMC3704572

[B116] Suadicani S. O. Brosnan C. F. Scemes E. (2006). P2X7 receptors mediate ATP release and amplification of astrocytic intercellular Ca2+ signaling. J. Neurosci. 26 (5), 1378–1385. 10.1523/JNEUROSCI.3902-05.2006 16452661 PMC2586295

[B117] Suarez-Berumen K. Collins-Hooper H. Gromova A. Meech R. Sacco A. Dash P. R. (2021). Pannexin 1 regulates skeletal muscle regeneration by promoting bleb-based myoblast migration and fusion through a novel lipid based signaling mechanism. Front. Cell Dev. Biol. 9, 736813. 10.3389/fcell.2021.736813 34676213 PMC8523994

[B118] Tai Y. Wang Q. Korner H. Zhang L. Wei W. (2018). Molecular mechanisms of T cells activation by dendritic cells in autoimmune diseases. Front. Pharmacol. 9, 642. 10.3389/fphar.2018.00642 29997500 PMC6028573

[B119] Taylor K. A. Wright J. R. Vial C. Evans R. J. Mahaut-Smith M. P. (2014). Amplification of human platelet activation by surface pannexin-1 channels. J. Thromb. Haemost. 12 (6), 987–998. 10.1111/jth.12566 24655807 PMC4238786

[B120] Thompson R. J. MacVicar B. A. (2008). Connexin and pannexin hemichannels of neurons and astrocytes. Channels 2 (2), 81–86. 10.4161/chan.2.2.6003 18849665

[B121] Thompson R. J. Zhou N. MacVicar B. A. (2006). Ischemia opens neuronal gap junction hemichannels. Science 312 (5775), 924–927. 10.1126/science.1126241 16690868

[B122] Vignery A. (2005). Macrophage fusion: The making of osteoclasts and giant cells. J. Exp. Med. 202 (3), 337–340. 10.1084/jem.20051123 16061722 PMC2213072

[B123] Wang J. Ambrosi C. Qiu F. Jackson D. G. Sosinsky G. Dahl G. (2014). The membrane protein Pannexin1 forms two open-channel conformations depending on the mode of activation. Sci. Signal. 7 (335), ra69. 10.1126/scisignal.2005431 25056878 PMC4243966

[B124] Wang J. Ma M. Locovei S. Keane R. W. Dahl G. (2007). Modulation of membrane channel currents by gap junction protein mimetic peptides: Size matters. Am. J. Physiol. Cell Physiol. 293 (3), C1112–C1119. 10.1152/ajpcell.00097.2007 17652431

[B125] Weilinger N. L. Lohman A. W. Rakai B. D. Ma E. M. Bialecki J. Maslieieva V. (2016). Metabotropic NMDA receptor signaling couples Src family kinases to pannexin-1 during excitotoxicity. Nat. Neurosci. 19 (3), 432–442. 10.1038/nn.4236 26854804

[B126] Weilinger N. L. Tang P. L. Thompson R. J. (2012). Anoxia-induced NMDA receptor activation opens pannexin channels via Src family kinases. J. Neurosci. 32 (36), 12579–12588. 10.1523/JNEUROSCI.1267-12.2012 22956847 PMC6621249

[B127] Willebrords J. Maes M. Crespo Yanguas S. Vinken M. (2017). Inhibitors of connexin and pannexin channels as potential therapeutics. Pharmacol. Ther. 180, 144–160. 10.1016/j.pharmthera.2017.07.001 28720428 PMC5802387

[B128] Woehrle T. Yip L. Elkhal A. Sumi Y. Chen Y. Yao Y. (2010). Pannexin-1 hemichannel-mediated ATP release together with P2X1 and P2X4 receptors regulate T-cell activation at the immune synapse. Blood 116 (18), 3475–3484. 10.1182/blood-2010-04-277707 20660288 PMC2981474

[B129] Wu C. H. Liu I. J. Lu R. M. Wu H. C. (2016). Advancement and applications of peptide phage display technology in biomedical science. J. Biomed. Sci. 23, 8. 10.1186/s12929-016-0223-x 26786672 PMC4717660

[B130] Xiong X. X. Gu L. J. Shen J. Kang X. H. Zheng Y. Y. Yue S. B. (2014). Probenecid protects against transient focal cerebral ischemic injury by inhibiting HMGB1 release and attenuating AQP4 expression in mice. Neurochem. Res. 39 (1), 216–224. 10.1007/s11064-013-1212-z 24317635

[B131] Yang D. He Y. Munoz-Planillo R. Liu Q. Nunez G. (2015). Caspase-11 requires the pannexin-1 channel and the purinergic P2X7 pore to mediate pyroptosis and endotoxic shock. Immunity 43 (5), 923–932. 10.1016/j.immuni.2015.10.009 26572062 PMC4795157

[B132] Yang Y. Delalio L. J. Best A. K. Macal E. Milstein J. Donnelly I. (2020). Endothelial pannexin 1 channels control inflammation by regulating intracellular calcium. J. Immunol. 204 (11), 2995–3007. 10.4049/jimmunol.1901089 32312847 PMC7336877

[B133] Yi K. S. Chung J. H. Lee Y. H. Chung H. G. Kim I. J. Suh B. C. (2001). Inhibition of the EGF-induced activation of phospholipase C-gamma1 by a single chain antibody fragment. Oncogene 20 (55), 7954–7964. 10.1038/sj.onc.1204959 11753678

[B134] Zerr M. Hechler B. Freund M. Magnenat S. Lanois I. Cazenave J. P. (2011). Major contribution of the P2Y₁receptor in purinergic regulation of TNFα-induced vascular inflammation. Circulation 123 (21), 2404–2413. 10.1161/CIRCULATIONAHA.110.002139 21576651

